# Characterization and Functional Analysis of *GhNAC82*, A NAM Domain Gene, Coordinates the Leaf Senescence in Upland Cotton (*Gossypium hirsutum* L.)

**DOI:** 10.3390/plants11111491

**Published:** 2022-06-01

**Authors:** Chenlei Wang, Tengyu Li, Qibao Liu, Libei Li, Zhen Feng, Shuxun Yu

**Affiliations:** 1 The Key Laboratory for Quality Improvement of Agricultural Products of Zhejiang Province, College of Advanced Agricultural Sciences, Zhejiang Agriculture and Forestry University, Hangzhou 311300, China; wangchenlei18@163.com (C.W.); libeili@zafu.edu.cn (L.L.); 2 State Key Laboratory of Cotton Biology, Institute of Cotton Research of the Chinese Academy of Agricultural Sciences, Anyang 455000, China; tengyuli18@163.com (T.L.); liuqibao566@163.com (Q.L.)

**Keywords:** cotton, *GhNAC82*, leaf senescence, abiotic stress, phytohormone-treated

## Abstract

In the process of growth and development, cotton exhibits premature senescence under various abiotic stresses, impairing yield and fiber quality. NAC (NAM, ATAF1,2, and CUC2) protein widely distributed in land plants, play the critical role in responding to abiotic stress and regulating leaf senescence. We have identified and functional analyzed a NAM domain gene *GhNAC82* in upland cotton, it was located on the A11 chromosome 4,921,702 to 4,922,748 bp, only containing one exon. The spatio-temporal expression pattern analysis revealed that it was highly expressed in root, torus, ovule and fiber development stage. The results of qRT-PCR validated that *GhNAC82* negatively regulated by salt stress, drought stress, H_2_O_2_ stress, IAA treatment, and ethylene treatment, positively regulated by the ABA and MeJA treatment. Moreover, heterologous overexpression of *GhNAC82* results in leaf premature senescence and delays root system development in *Arabidopsis thaliana*. The phenotype of delayed-senescence was performed after silencing *GhNAC82* by VIGS in premature cotton. Taken together, *GhNAC82* was involved in different abiotic stress pathways and play important roles in negatively regulating leaf premature senescence.

## 1. Introduction

Leaf senescence, an internal factor, is the adaptive mechanism across long evolutionary distances and it is a prevalent phenomenon during plant development [1]. In addition, it is usually taking place in the final stage of plant growth, accompanied by complex and highly coordinated gene regulatory networks [2]. The phenomenon of plants emerges advanced chlorophyll degradation, the photosynthesis rate decreases, the accumulation of reactive oxygen species (ROS), and oxidative enzyme activity reduced in the appropriate growing season, termed premature senescence [3]. The occurrence of premature aging is the result of a combination of multiple factors. Extreme environmental conditions adversely affect the growth of plants and accelerate leaf senescence, including drought, salt, pathogenic bacteria, and osmotic stress, which seriously restricts crop yield and quality [4,5,6,7]. Otherwise, genetic factors also play a crucial role, such as gene mutations leading to the premature-aging. The regulation of leaf senescence involves the dynamic changes of related gene expression and protein synthesis, and the transcription factors (TFs) and the downstream gene promoters cis-elements are combined to regulate the senescence process [8,9,10]. Transcriptional regulation plays a critical role in plant maturation and senescence, and TFs mediate senescence regulatory networks by activating or inhibiting target genes [11].

NAC is a family of plant-specific transcription factors that regulate the normal growth and development of plants under various abiotic stress response and aging process [12,13]. All of the N-terminals of their encoding proteins contain a conserved sequence of about 150 amino acid residues, which plays an important role in plant growth and development [14]. It could be degraded by protease or ubiquitin-proteasome under specific environmental signals, and the homologous or heterodimer of the activated NAC protein combines with the promoter of its target gene to control its expression during the nuclear signal input [15,16]. Overexpression of *OsNAC9* and *OsNAC10* regulates root architecture to enhance drought resistance and increase the grain yield of rice [17,18]. Similar results were found in *Arabidopsis*, *AtNAC1* and *AtNAC2* are induced by auxin to promote lateral root elongation, and both *AtNTL8* and *AtLOV1* overexpressed showed slow growth and delayed flowering [19,20,21,22]. Involvement of *ANAC003*, *ANAC010*, *ANAC042*, and *ANAC075* under dark stress tolerance was corroborated by the phenotype of *Arabidopsis tic55-II* knock-out (KO) mutants and found the promoter region of *ANAC003* bound to MYB108 in yeast one-hybrid (Y1H), taking into account the above indicate that the regulation network of MYB-NAC is involved in the process of aging under dark stress [23]. *ANAC072* positively regulates natural and dark-induced leaf senescence through chlorophyll degradation and activating the transcription of NYE1, which is induced by multiple stresses [24].

NAC as a regulatory factor of leaf senescence has been confirmed in the major crops and the regulatory network can be mediated by changes in endogenous hormone levels [25,26,27]. Phytohormones play a key role in response to biotic and abiotic stresses and have been found to involved in the regulation of leaf senescence; specifically, indole-3-acetic acid (IAA), cytokinins (CTK), and gibberellin (GA) are thought to delay leaf senescence, whereas ethylene (ETH), abscisic acid (ABA), methyl jasmonate (MeJA), salicylic acid (SA), brassinosteroids (BR) are thought to induce it. A previous study reported that the content of IAA gradually increases in *Arabidopsis* leaves during later development stages, indicating auxin is involved in the regulation mechanism of senescence [28]. The content of endogenous cytokinins decreased promotes cell division synergistically with auxin to delay leaf senescence, and cytokinin-mediated *AtAHK3* controls aging by phosphorylating *AtARR2* [29,30]. The root system also affects leaf senescence in *G. hirsutum* directly or indirectly via increased the biosynthesis and transport of abscisic acid [31]. Exogenous treatment of methyl jasmonate reduces plant growth in rice by decreasing chlorophyll contents and photosynthetic capacity and induces the expression of *OsDOS* genes [32]. Plant hormones respond to signaling responses to biotic and abiotic stresses by interacting with or antagonizing NAC transcription factors and their upstream or downstream genes to maintain the stability of the intracellular environment [33]. Likewise, exogenous treatment of plant hormones inducible expression of NAC transcription factor, and the ATAF subfamily positive function in hormones-dependent stress-response pathways, *ANAC019* and *ANAC055* are negative regulators of stress [18,34].

The completion of genome sequencing has laid the foundation for the research on the regulation of leaf senescence by cotton NAC transcription factors at the genome level [35,36]. Several upland cotton NAC genes (such as *GhNAC2*, *GhNAC4*, and *GhNAC6*) have been isolated in response to abiotic stresses: drought, salt, high and low temperatures by the ABA signal transduction pathway [37,38]. Overexpression of *GhNAC79* enhanced the expression level in the cotyledon and fiber development stage significantly improved the resistance to drought stress of upland cotton and *A. thaliana* [39]. It has been found that seven genes in upland cotton (*GhNAC7*-*GhNAC13*) were highly expressed in roots indicating these genes might be involved in responses to abiotic stress during root development [40]. In addition, *GhNAC63* and *GhNAC78* have already been identified to involved in the regulation of leaf senescence [41,42]. These results all indicated that *NAC* gene family members play an essential role in the regulation of leaf senescence, stress resistance, and phytohormone-responsive in cotton. Therefore, it is necessary to unravel the conduction mechanisms of NAC genes in aspects of stress-responsive and leaf senescence, which further improves crop yield and quality.

In this study, we revealed the function and molecular mechanism of *GhNAC82* in upland cotton leaf senescence. The expression patterns of *GhNAC82* in response to different abiotic stresses and hormonal treatments were analyzed, and the results showed that it is particularly sensitive to changes imposed by exogenous hormones. The overexpression of *GhNAC82* in *A. thaliana* and silence in differentially aging cotton shows that overexpress led to premature aging, silence delayed leaf senescence. These results show the function of *GhNAC82* in vegetative growth, and it will provide the theoretical basis for the breeding of new cotton varieties with early maturity but no premature senescence.

## 2. Materials and Methods

### 2.1. Plant Materials, Growth Conditions and Stress Treatments

One the premature senescence upland cotton (cv. CCRI 10) and normal senescence upland cotton (cv. CCRI 50) were used in this study. Plump seeds were selected and cultivated in seedlings pot and grew up in an artificial climate box (16 h light 28 °C/8 h dark 25 °C cycle), normal water and fertilizer management was administrated until the end of the experiment.

Select seedlings with consistent growth for the first sampling when cotyledons spread out. Then the samples were taken every one week to observe the senescence change of the cotyledons until the cotyledons turn completely aged after seven weeks. Selecting the root, stem, leaf, torus, petals, stamen, and pistil form healthy plants, quickly stored in liquid nitrogen. Samples of ovules and fibers were taken at 0, 5, 10, 15, 20, and 25 DPA (days post anthesis), respectively. After the unfolding of the third leaf, seedlings were irrigated with 20% PEG 6000 or 200 mM NaCl at 5-day intervals and sampling at 0, 5, 10, 15, 20, and 25 days after drought/salt treatment. For hormone treatment at the same period, seedlings were sprayed with 20 µM auxin, 50 µM ABA, 200 µM MeJA, and 200 µM ethephon, and controlled with water. The leaf samples were collected at 0, 4, 12, 24, and 48 h after treatment. All of the above samples contain at least 3 biological repetitions.

*A. thaliana* ecotype *Col-0* (wild-type) was used as the heterologous transformation recipient. After seeds are vernalized, planted in an incubator at 16 h light (25 °C)/8 h dark (22 °C).

### 2.2. Gene Cloning and Sequence Analysis

cDNA sequence of *GhNAC82* (1047 bp, GenBank accession number: KF669793) was cloned from the leaf of CCRI 10. The basic information and physicochemical properties of the GhNAC82 were predicted by the smart program via the ExPASy Proteomics Server [43]. The gene structure was scaled by Gene Structure Display Server (GSDS, http://gsds.cbi.pku.edu.cn/, accessed on 10 May 2020). Nucleotide sequences of 2000 bp in upstream regions of *GhNAC82* were obtained from CottonFGD [44], while performing cis-acting elements analysis by PlantCare [45]. Multi-sequence alignments and phylogenetic analyses of *Arabidopsis*, *G. arboreum*, *G. raimondii*, *G. hirsutum*, *G. barbadense*, cocoa, soybean, grape, and sorghum NAC proteins were carried out with the ClustalW [46], phylogenetic tree based on the alignment using the method of Neighbor-Joining by MEGA 7.0 (bootstrap replications set to 1000) [47]. Chromoal localization is illustrated using the MG2C v2.1 (http://mg2c.iask.in/mg2c_v2.0/, accessed on 11 May 2020) based on the specific location of *GhNAC82* on the genome. RNA-Seq data was downloaded from Cotton Omics Database (http://cotton.zju.edu.cn/10.rnasearch, accessed on 10 June 2021) to analyze *GhNAC82* under different tissues and abiotic stresses (salt, PEG, low and high temperature), the TBtools to visualize it [36,48].

### 2.3. DNA, RNA Extraction, cDNA Synthesis, and Quantitative RT-PCR (qRT-PCR)

The DNA of *Arabidopsis* was extracted from the TIANGEN plant genome DNA extraction kit (centrifugal column type, DP305), and the DNA of upland cotton was extracted using the plant DNA pickup kit (YK00A5). RNA extraction was following the manufacturer’s protocol for plants rich in polyphenols and amylase total RNA extraction kit (Vazyme, RC401, Nanjing, China), the quality of all samples was detected by 1% agarose gel electrophoresis and A260/A280 values by NanoDrop 2000. cDNA synthesis according to the Vazyme reverse transcription kit instructions (Vazyme HiScript II 1st Strand cDNA Synthesis Kit, R212).

Quantitative RT-PCR used ChamQ universal SYBR qPCR Master Mix (Vazyme, Q711), with *GhUBQ7* as the reference gene. The running procedure was established as described in the manual, the expansion curve and dissolution curve of real-time PCR were confirmed after the reaction, and the results were analyzed using 2^−∆∆Ct^ data [49]. The entire trials were performed under low-light conditions, each data contains three technical repetitions and three biological repetitions at least. All primer sequences are listed in Table S1.

### 2.4. Vector Construction and Genetic Transformation of A. thaliana

PCR amplification products of *GhNAC82* were inserted into the EcoRI and XbaI sites of the binary vector pBI121 to generate *35S::GhNAC82* plasmids, and the *35S::GhNAC82* recombinant plasmid was introduced into *A. thaliana* wild type (*Col-0*) by the *Agrobacterium* (GV3101). Positive plants were selected on 1/2 MS media containing kanamycin (100 mg/L), and the green plants were transplanted in the seedling pot and further confirmed by PCR and qRT-PCR after 14 days.

### 2.5. Viruses Induced GhNAC82 Gene Silencing in Cotton

The *PYL156*, *PYL192*, *PYL*::*GhPDS* silencing systems induce *GhNAC82* silencing in cotton [50]. The fragments of *GhNAC82* were inserted into the *PYL156* vectors with EcoRI and KpnI cutting sites using homologous recombination to construct *PYL156*::*GhNAC82*. *PYL156*, *PYL*::*GhPDS*, *PYL192*, and inserted fragment vectors were transformed into *Agrobacterium tumefaciens* (GV3101), then collected and resuspended in infiltration buffer (10 mM MgCl_2_, 10 mM MES, and 200 μM acetosyringone). The concentration of the cell suspension was set to 0.8–1.2 (OD600). *PYL156*, *PYL156*::*GhPDS*, and *PYL156*::*GhNAC82* derivatives were mixed with *PYL192* in equal proportions and kept from light for 4 h at room temperature. Cotton seedlings were grown in an artificial climate box (16 h light 25 °C/8 h dark 23 °C cycle).

### 2.6. Determination of the Related Physiological Parameters

The malondialdehyde (MDA) contents were quantified from 100 mg cotton leaves using an assay kit as the instructions described (Solarbio, BC0025, Beijing, China). The chlorophyll content was determined according to the method as the previous study described [51]. The soluble protein was determined according to the method described by Enzyme Free Company Plant Soluble Protein (sprotein) ELISA kit and Coomassie brilliant blue G250 method.

## 3. Results

### 3.1. Identification and Evolution Analysis of the GhNAC82

NAC transcription factor family exerted a vital regulatory effect on leaf senescence, stress resistance, and phytohormone-responsive in cotton. *GhNAC82* was selected for further functional studies. *GhNAC82* contained only one exon, the coding sequences (CDS) with the same nucleotide composition as genomic DNA (gDNA) sequences, and it was isolated from the cotton variety CCRI 10. The full-length cDNA includes a 1047 bp open reading frame ORF encoding a 348 amino acid protein with a molecular weight (MW) is 38.727 kDa. It was found to be localized on chromosome A11: 4,921,702–4,922,748 bp.

**Figure 1:**
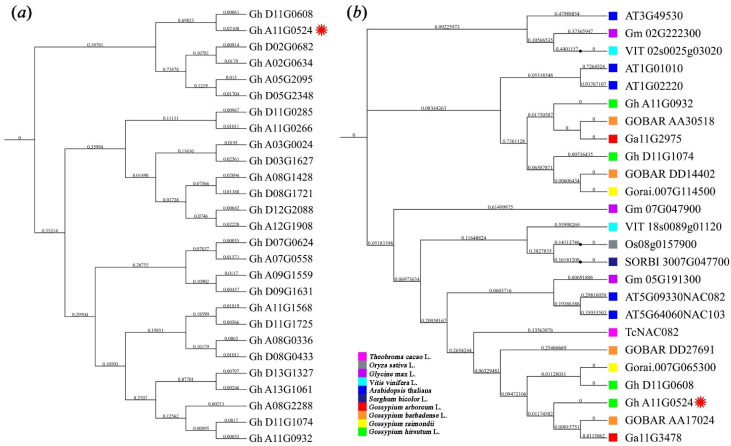
Evolution of *GhNAC82* in upland cotton (**a**) and other species (**b**). red * indicated the GhNAC82 gene.

According to the domain scrutiny and relationships analysis, 27 proteins were identified from upland cotton (Figure 1a). Of these, the closest relative to *GhNAC82* (*Gh_A11G0524*) was another copy on the D subgroup (*Gh_D11G0608*), followed by *GhNAC17* (*Gh_D02G0682*, *Gh_A02G0634*, *Gh_A05G2095*, *Gh_D05G2348*). Physicochemical properties and gene information of these NAC proteins in *G. hirsutum* are presented in Table S2.

With MEGA 7.0 software construct a phylogenetic tree from the aligned sequences, showing evolutionary relationships between *GhNAC82* and other homologous genes from upland cotton and representative plant species (including *G. arboreum*, *G. raimondii*, *G. hirsutum*, *G. barbadense*, *A. thaliana*, *Theobroma cacao*, *Oryza sativa*, *Glycine max*, *Vitis vinifera*, and *Sorghum bicolor*, Figure 1b). The phylogenetic analysis characterized the closest evolutionary relationship to *GhNAC82* (*Gh_A11G0524*) were *GOBAR_AA17024* from *G. barbadense* A subgenome and *Ga11G347* from the diploid ancestor *G. arboreum*. Meanwhile, the closest evolutionary relationship to *Gh_D11G0608* (another homologous gene in D genome of upland cotton) was *GOBAR_DD27691* from *G. barbadense* D subgenome and *Gorai.007G065300* from the diploid ancestor *G. raimondii*. In the evolutionary between cotton and other representative species, the closest phylogenetic relationship was *T. cacao* (*TcNAC082*), secondly is *A. thaliana* (*AT5G09330* and *AT5G64060*), followed by *G. max* (*Gm_05G191300*), *S. bicolor* (*SORBI_3007G047700*), *O. sativa* (*Os08g0157900*), and *V. vinifera* (*VIT_18s0089g01120*). These genes may have a similar biological function to *GhNAC82*, and might be the connection to leaf senescence and stress tolerance.

### 3.2. Predicted of Cis-Elements in Promoter Region of GhNAC82

**Figure 2:**
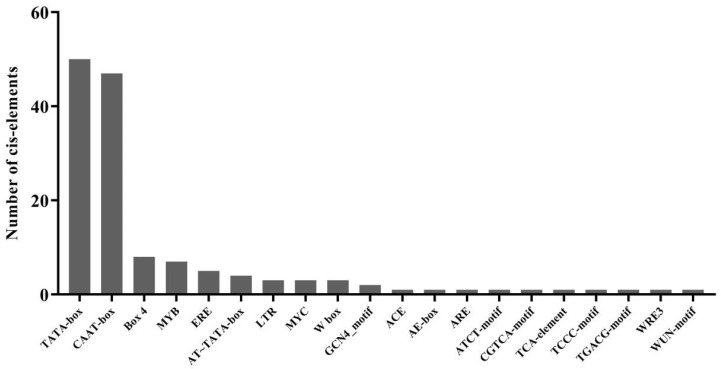
Statistics of cis-elements in promoters of *GhNAC82*.

The cis-regulatory elements in the promoter usually regulate gene expression and respond to environmental and growth factors [52]. Cis-acting regulatory elements were analyzed in the *GhNAC82* upstream 2000 bp promoter region by bioinformatics using the PlantCARE database. As shown in Figure 2, the promoter sequence of *GhNAC82* contains the basal cis-elements TATA-box and CAAT-box. The cis-elements involved in plant growth and development, stress response, and hormone response were found. Among these cis-elements, including the light responded elements: such as ACE, AE-box, ATCT-motif, Box 4, MRE, and TCCC-motif. Furthermore, the binding site of AT-rich DNA binding protein (ATBP-1, AT-rich element), endosperm expression elements (GCN4_motif), and the anaerobic induction element (ARE) was found to be related to plant growth and development. It was also found that the stress-related elements contained low-temperature responsiveness (LTR). Phytohormone correlated elements include MeJA (CGTCA-motif, TGACG-motif), gibberellin (GA, TATC-box), and salicylic acid (SA, TCA-element). In addition, MYC and MYB-binding sites involved in dehydration-responsive, the cis-elements involved in wound-responsiveness, such as W-box, WRE3, and WUN-motif (Figure 2, Table S3). These results suggest that the *GhNAC82* may be involved in the stress responses of plant growth and stress.

### 3.3. Expression Patterns Analysis of GhNAC82 in Various Tissues

Spatiotemporal expression files of *GhNAC82* were appraised in seven different tissues (including root, stem, leaf, torus, petals, stamen, and pistil) and development period (including 0-, 5-, 10-, 20-, and 25-DPA ovule; 10-, 20-, and 25-DPA fiber) using RNA-seq data from *G. hirsutum*. It was found that *GhNAC82* was highly expressed in root, petals, 10 DPA ovule and fiber, 20 DPA fiber, and 25 DPA fiber. Secondly, the lower expression in torus, 5 DPA ovule, and 20 DPA ovule. The results of the gene expressed in ovule and fiber suggested that it may play a key role in regulating expression during embryonic and fiber development.

**Figure 3:**
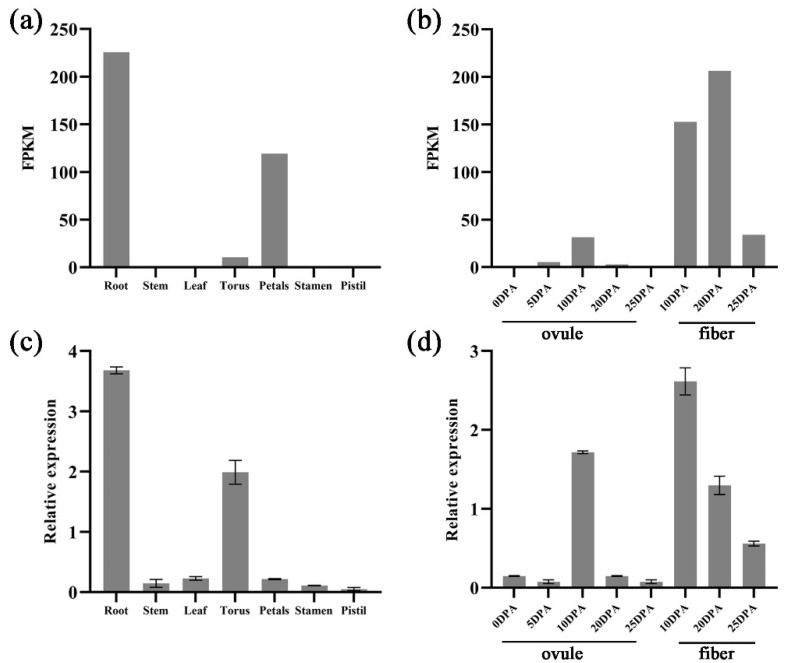
Expression patterns of *GhNAC82* in different tissues and development periods. (**a**,**b**) The expression patterns of *GhNAC82* in different tissues were plotted according to FPKM (Fragments Per Kilobase per Million mapped reads) values; (**c**,**d**) The expression patterns of *GhNAC82* in various tissues and different stages of ovule and fiber development were plotted according to the results by quantitative real-time PCR.

To detect the expression patterns of *GhNAC82* in different tissues, the roots, stems, and leaves in the seedling stage; torus, petals, stamen, and pistil in the flowering period were collected. At the same time, ovules in 0 DPA, 5 DPA, 10 DPA, 20 DPA, 25 DPA, and fibers in 10 DPA, 20 DPA, and 25 DPA were collected. The pattern of gene expression assessed by qPCR was basically consistent with transcriptome profiling data, as shown in Figure 3. However, it demonstrated different patterns, it was found that *GhNAC82* was expressed in stem, leaf, stamen, pistil, 0 DPA ovule, and 25 DPA ovule using real-time PCR. Overall, these results indicated that *GhNAC82* may play a critical regulatory role in root growth and development.

### 3.4. Phenotypic Changes and Expression Patterns of GhNAC82 during Cotyledon Senescence

**Figure 4:**
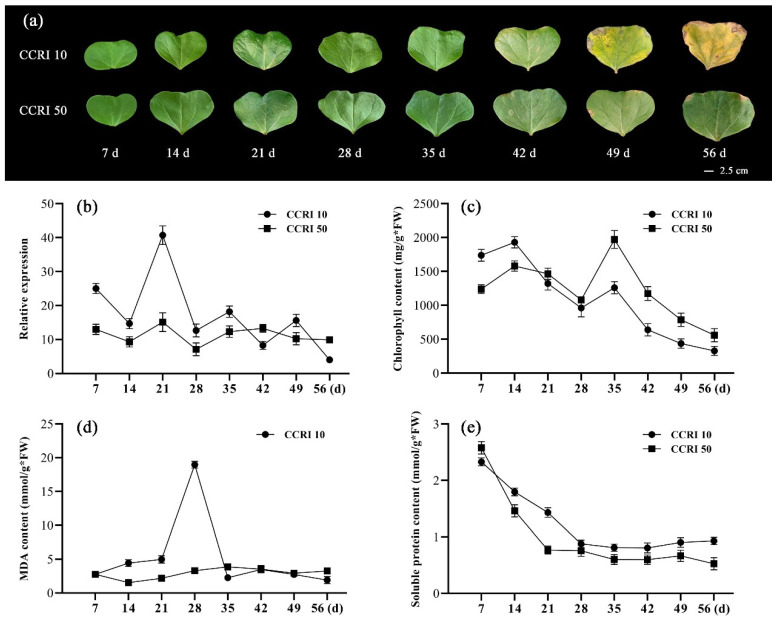
Expression profiles and physiological indexes in 7-, 14-, 21-, 28-, 35-, 42-, 49-, and 56-day-old of the CCRI 10 and CCRI 50 cotton variety during cotyledon senescence. (**a**) Colors change of cotyledon during developmental stages; (**b**) The expression pattern of *GhNAC82* during cotyledon development, and *GhUBQ7* was used as reference gene; (**c**–**e**) The content of chlorophyll, MDA, and soluble protein among different developmental stages, *n* ≥ 3.

During the leaf grew and developed, chlorophyll contents and photosynthetic efficiency were gradually decreased as mature leaves yellowing. As shown in Figure 4a, with the growth and development, cotyledon color was progressively changed and CCRI 10 was significantly higher than CCRI 50 based on teh degree of yellowing. The cotyledon of CCRI 10 was obviously yellowing on 49 days, completely yellow even withered on 56 days, while the CCRI 50 only a small section of the cotyledon was with brown spots or shrinking. Figure 4b shows that *GhNAC82* was highest expressed in the 21 days after cotyledons unfolding of CCRI 10. Subsequently, the expression gradually decreased. From the patterns of *GhNAC82* during cotyledon senescence, we presumed that *GhNAC82* likely has an important role in the early cotyledon development stage. Differing from the patterns in CCRI 10, the expression of *GhNAC82* was relatively gentle in CCRI 50, suggesting that elevated *GhNAC82* expression may be responsible for premature senescence.

The content of chlorophyll, malonaldehyde (MDA), and soluble protein are the integral part and indicators of the degree of leaf senescence [53]. Chlorophyll is the main pigment for photosynthesis and is closely related to the aging process in plant growth and development. Malondialdehyde (MDA) is the oxidation final product of free radicals acting on the lipid with peroxidation reaction in the organism, which is cytotoxic and is closely related to the stress-related response [54]. Soluble protein content increased in the stressed seedlings stage, and it also was a key abiotic stress indicator in plants [55]. As Figure 4c shows, the chlorophyll content has peaked on 14 and 35 days of cotyledon development, with an overall, showed downward trend. In addition, the chlorophyll in CCRI 50 exhibited higher content than CCRI 10 after 21 days. The MDA content in CCRI 10 and CCRI 50 showed a uniform upward trend, then the MDA content of CCRI 10 is peaked at 28 days, and CCRI 50 is peaked at 35 days but the increasing trend was relatively flat in CCRI 50. After reaching the peak, followed by a slow downward trend (Figure 4d). The content of soluble protein in CCRI 10 always remained higher than CCRI 50 after 14 days, and it showed two peaks at 21 days and 35 days (Figure 4e). It was coincided with the relative expression of *GhNAC82* and chlorophyll content among different cultivars and indicated the content of soluble protein is strongly correlated with the degree of leaf senescence. Both results suggest that probably exist a potential regulatory mechanism is via the expression of *GhNAC82* mediates the physiological indicators to regulate the leaf senescence.

### 3.5. Expression of the GhNAC82 in Leaves Was Significantly Induced by Abiotic Stresses

**Figure 5:**
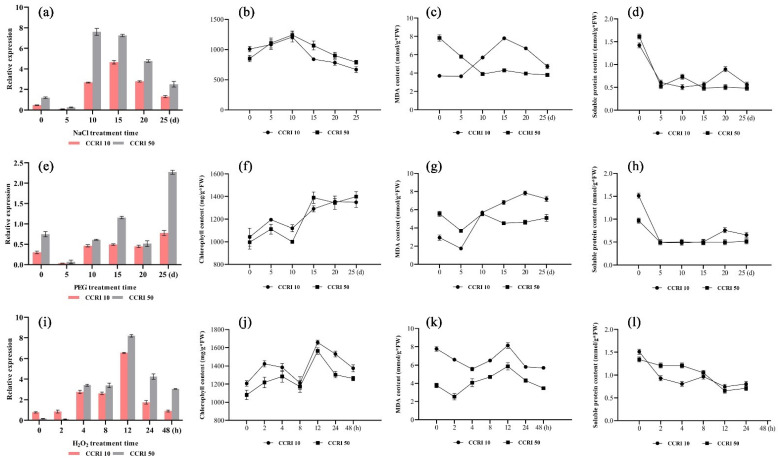
Expression patterns and related physiological metrics changes of *GhNAC82* after salt, drought, and H_2_O_2_ stress. (**a**,**e**,**i**) Expression pattern of *GhNAC82* under NaCl, PEG, and H_2_O_2_ treatment; (**b**,**f**,**j**) Changes of chlorophyll content under treatments; (**c**,**g**,**k**) Changes of malondialdehyde content under treatments; (**d**,**h**,**l**) Changes of soluble protein content under treatments.

To investigate whether are responsive regulatory proteins under abiotic stresses in different degrees of prematurity species, NaCl, PEG, and H_2_O_2_ treatment of the CCRI 10 and CCRI 50 cotton seedlings were performed. As shown in Figure 5a,e,i, the expression levels of *GhNAC82* significantly changed after salt, drought, and H_2_O_2_ treatment, and the expression in CCRI 50 was higher than in CCRI 10 all the time (except for the early H_2_O_2_-stress, 0 h and 2 h).

The two cotton species (phenotype of premature senescence and non-premature senescence) showed a peak at the time 10 days after treatment and gradually decreased subsequently. A similar trend was observed in the changes in chlorophyll contents with expression pattern (Figure 5b). Malondialdehyde content was increased gradually after the peak time of 10 days (Figure 5c). After drought stress, the expression of *GhNAC82*, chlorophyll and MDA contents exhibited a continuously rising trend (Figure 5e–g). H_2_O_2_-treated plants show expression peaked at 12 h, and decline followed (Figure 5i). Chlorophyll content in leaves under H_2_O_2_ stress reached a maximum in 12 h too (Figure 5j). MDA contents peaked relatively early under the H_2_O_2_ treatment condition, and it appeared to maximum advanced one stage (at 8 h, Figure 5k). All the soluble protein content under abiotic stress conditions exhibit decreased slowly with treatment time (Figure 5d,h,l). From the above results, we can conclude that the *GhNAC82* gene is involved in multiple abiotic stress responses, and its’ expression may be negatively regulated by salt, drought, and H_2_O_2_ treatment. In addition, chlorophyll and MDA content are tightly linked to the expression of *GhNAC82*, soluble protein content showed a dynamic decrease as the abiotic stress progressed.

### 3.6. The Expression Patterns of GhNAC82 under Different Hormones

Different hormone treatments were performed for cotton seedlings at CCRI 10 and CCRI 50, respectively, and the expression of GhNAC82 was detected during different treatment periods. The expression level of *GhNAC82* changed significantly in response to phytohormone treatments.

**Figure 6:**
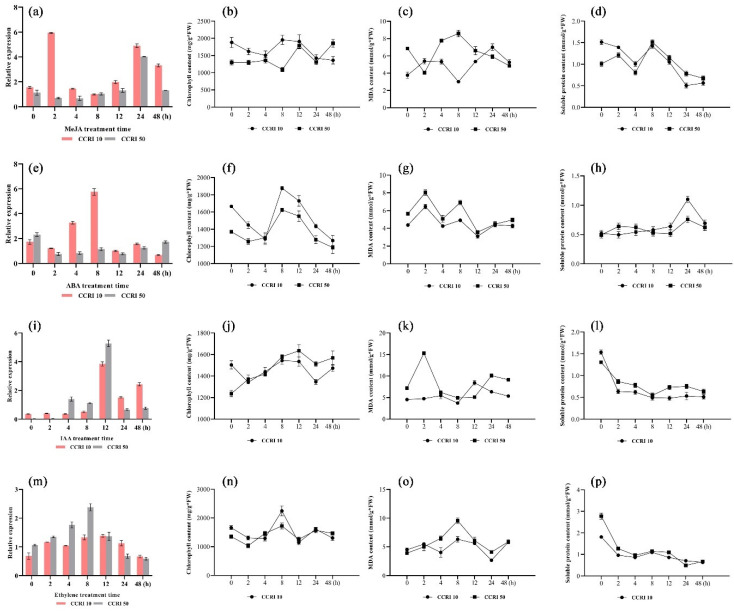
Expression patterns and physiological indicators of *GhNAC82* in response to phytohormone treatments. (**a**,**e**,**i**,**m**) Expression pattern of *GhNAC82* under treatment with auxin, abscisic acid, ethylene and methyl jasmonate; (**b**,**f**,**j,n**) Changes of chlorophyll content under phytohormone treatments; (**c**,**g**,**k,o**) Changes of malondialdehyde content under phytohormone treatments; (**d**,**h**,**l,p**) Changes of soluble protein content under phytohormone treatments.

In the MeJA treatment initial periods (2 h after treatment), there was induction reaching a significance difference, and the expression of *GhNAC82* in CCRI 10 increased to approximately 3 times. After that, the expression falls rapidly, 24 h a second peak was reached (Figure 6a). However, only the expression peaked at 24 h, and the expression was lower than CCRI 50 all the time. A trend of first rising and then falling was seen in soluble protein content, but the content of chlorophyll and malondialdehyde did not show a clear trend (Figure 6b–d).

The expression pattern of *GhNAC82* in CCRI 10 demonstrated similar trends under ABA treatment compared with MeJA treatment, and it was peaked at 8 h after treatment (Figure 6e). The content of chlorophyll and malondialdehyde also reached a peak at 8h after treatment (Figure 6f,g). Although the soluble content was on the rise and peaked at 24 h, there is no obvious trend (Figure 6h).

In contrast to the expression trend in MeJA and ABA response, the expression levels under IAA and ethylene treatment of *GhNAC82* in CCRI 50 exhibited higher than in CCRI 10. This pattern of expression is similar to that described in abiotic stresses (Figure 5). Meanwhile, a peak expression under IAA and ethylene at 12 h and 8 h, respectively (Figure 6i,m). The content of chlorophyll and MDA also showed a trend of the first decline and then rise, timing to reach peak emergence broadly matched with the expression peak appeared (Figure 6j,k,n,o). The trend of changes in soluble protein is basically to decline (Figure 6l,p). These results all indicated that the induction expression of *GhNAC82* in phytohormone-treated, and was positively induced by MeJA and ABA treatment, negatively induced by IAA and ethylene treatment. Furthermore, the changes in chlorophyll and MDA content coincided with the expression patterns of *GhNAC82*. Additionally, the changes in soluble protein content basically displayed a decreasing trend under various hormone treatments.

### 3.7. Overexpression of GhNAC82 in Transgenic Arabidopsis Promotes Leaf Senescence

**Figure 7:**
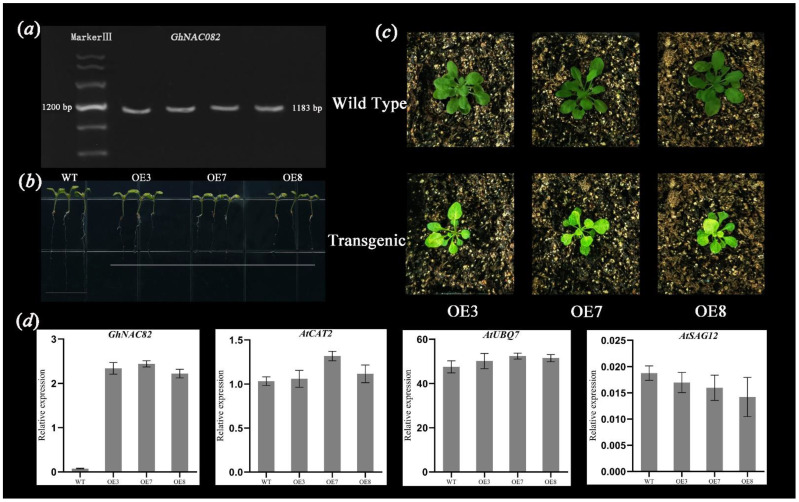
Overexpression of *GhNAC82* leads to premature senescence of *Arabidopsis* plants. (**a**) Agarose gel electrophoresis detection; (**b**) Comparison of root systems between wild-type and overexpression plants; (**c**) Comparison of leaf senescence degree between wild-type and overexpression plants; (**d**) Expression levels of *GhNAC82*, *AtCAT2*, *AtUBQ7*, and *AtSAG12* in wild-type and overexpression plants.

According to the agarose gel electrophoresis (Figure 7a), showed a high band brightness, and the length of the cloned *GhNAC82* gene CDS was 1047 bp, consistent with the sequencing gene CDS fragment size. Under normal growth conditions, the expression levels of senescence-related marker genes involved in wild type and overexpressed *GhNAC82* transgenic *A. thaliana* were detected, respectively. The internal reference primers (*AtActin2*) and other primers were presented in Table S1. As shown in Figure 7b, the overexpression-*GhNAC82* transgenic plants were exhibited a shorter root length. According to the leaf senescence phenotype characterized by the wild type and transgenic type *Arabidopsis*, GhNAC82-overexpressing plants showed an earlier senescence phenotype than the wild-type ones (Figure 7c). The expression level of the *GhNAC82* gene in the overexpressed transgenic plants was significantly higher than that in the wild type. In addition, the expression of *AtUBQ7* and *AtCAT2* (senescence-related gene) was higher than that in the wild type too, but the expression of *AtSAG12* was lower than wild type (Figure 7d). The data above indicate that the transgenic type plants’ root system grew slowly and promoted early leaf senescence compared with the wild type.

### 3.8. Virus-Induced Gene Silencing of GhNAC82 Exhibited Stronger Vegetative Growth

**Figure 8:**
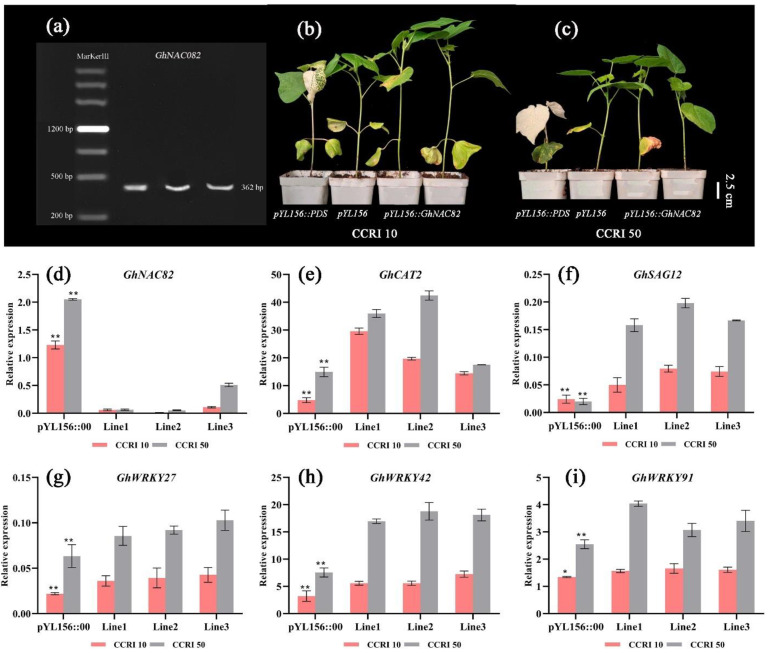
Phenotype and related gene expression of *GhNAC82*-silenced. (**a**) Agarose gel electrophoresis detection; (**b**,**c**). Comparison of plant height among positive control, negative control and silenced plants in CCRI 10 and CCRI 50; (**d**–**i**) Expression of *GhNAC82*, *GhCAT2*, *GhSAG12*, *GhWRKY27*, *GhWRKY42* and *GhWRKY91* in negative control and silent plants. *: Significance at *p* < 0.05; **: Significance at *p* < 0.01.

The GhNAC82 gene was silenced in CCRI 10 and CCRI 50 using virus-induced gene silencing (VIGS). In addition, the expression level of *GhNAC82* was detected by qRT-PCR after the albino phenotype appeared on the leaves of positive control material *PYL156*::*GhPDS*. As shown in Figure 8, after the virus infected cotton seedlings, the expression level of the *GhNAC82* gene in both CCRI 10 and CCRI 50 decreased to 1/20-1/10 times of negative control. It was indicating that VIGS effectively inhibited the expression of *GhNAC82*. The expression levels of other senescence related marker genes in CCRI 50 were higher than that in CCRI 10, and the expression levels of *GhNAC82* in negative control plants were higher than in *GhNAC82*-silenced plants. The expression levels of other senescence-related genes in negative control plants were lower than those in silenced plants, which may be the result of other senescence genes playing more roles after silencing *GhNAC82*. Compared with negative control plants, *GhNAC82*-silenced expression increased plant height in CCRI 10, but there were no noticeable changes in CCRI 50.

## 4. Discussion

Abiotic stress affects the productivity of major agricultural crops worldwide. NAC transcription factor is the largest family of plant-specific transcription factors that play crucial roles in plant growth and developmental processes, withstand abiotic stress, and delayed senescence [56]. It is distribution widespread in bryophytes to dicots genome, received much attention as an important regulator in gene function and regulation of expression [57,58]. In our study, we identified the orthologous genes in *GhNAC82* upland cotton and other species, which found the closest phylogenetic relationship genes was from *T. cacao*, second, is *A. thaliana*, followed by *G. max*, *S. bicolor*, *O. sativa*, and *V. vinifera* (Figure 1). The relatedness among the paralogous gene pairs was similar to the phylogenetic relatedness between species. This illustrates that these genes may play similar roles to leaf senescence and abiotic stress response. Meanwhile, the *GhNAC82* promoter regions have multiple growth and development elements, abiotic stress-responsive elements, light- and phytohormone-responsive elements (Figure 2). The cis-elements of the upstream promoter at the gene transcription start site regulate the expression of *GhNAC82* in response to various abiotic stresses and hormone treatment.

We found that the expression of *GhNAC82* was significantly induced for abiotic stresses and hormone treatment, which were negatively regulated by salt stress, drought stress, H_2_O_2_ stress, MeJA, and ABA treatment, positively regulated by IAA and ethylene treatment (Figure 5 and Figure 6). This finding was in agreement with the previous study on other plants. Overexpression of *SiNAC1* in *Arabidopsis* regulated the abscisic acid (ABA) accumulation to promote natural leaf senescence [59]. Ectopic expression of *OsNAC2* accelerated leaf senescence by up-regulated the expression levels of the abscisic acid (ABA) biosynthesis genes (*OsNCED3* and *OsZEP1*) and abscisic acid catabolism gene OsABA8ox1 [60]. Similarly, *VviNAC33* has been noted to promote leaf senescence by inhibiting the relative expression of auxin transport-related gene *PIN1* [61]. Apart from the above, the contradictory results among stress treatments and some phytohormone treatments (i.e., salt and ethylene treatment). Plants respond and adapt to stress by upregulating ethylene signal as the negative feedback regulators, this is consistent with our findings under ethylene treatment.

Previously reported that the NAC transcription factors regulate the formation of lateral roots, *AtNAC2* promotes lateral root development and inhibited the primary root growth to mitigate damage in *A. thaliana* [62]. In our study, transcriptome data and qRT-PCR results showed that *GhNAC82* was highest expressed in root, suggesting that *GhNAC82* might be related to root system development to regulated abiotic stress response (Figure 3). In parallel, we have generated the transgenic *Arabidopsis* overexpressing *GhNAC82*, and transgenic plants demonstrated a shorter primary root compared to wild-type (Figure 7). This result is basically consistent with the results of the previous study in *Arabidopsis*, *Solanum tuberosum*, and *Zea mays* [59,63]. In addition, *AtORS1*-overexpression (*AtORS1*: NAC transcription factor) has been shown to promote premature senescence, and it was up-regulated the expression of multiple genes related to salt and H_2_O_2_ stress [64]. Combined the results of *GhNAC82*-silenced expression increased plant height and delayed senescence indicating *GhNAC82* is a negative regulator of leaf senescence. The combination of these results and our study illustrating NAC transcription factor family member *GhNAC82* plays an important role in the regulation of leaf senescence, hormone, and abiotic stresses responses.

Similar to in other plants, premature senescence in cotton is a complex polygenic trait, and it is linked to multiple signal transduction pathways. Based on available studies of premature senescence, regulating expression levels of NAC genes to achieve elite germplasm resources that are tolerant to multiple abiotic stresses using gene engineering method and genome wide association studies (GWAS) [65]. In parallel, investigating the molecular mechanisms of senescence to develop and grow normally. In summary, elucidation of the functions of *GhNAC82* has important implications to develop crop precision breeding.

## 5. Conclusions

In this study, we performed the protein sequences and evolutionary relationships analysis of *GhNAC82* and orthologous genes from upland cotton and other species by taking advantage of the published genome data to date. The expression patterns of *GhNAC82* under various tissues and development stages, different abiotic stresses, and phytohormone-treated were carried out using qRT-PCR. It was found that *GhNAC82* was higher expressed in root, torus, 10 DPA ovules, and fibers in 10 DPA, 20 DPA, and 25 DPA. In addition, these results indicate that *GhNAC82* is negatively regulated by salt stress, drought stress, H_2_O_2_ stress, IAA treatment, and ethylene treatment. Meanwhile, it is positively regulated by the ABA and MeJA treatment. Expression patterns change of *GhNAC82* in response to phytohormone treatments and abiotic stress, the relevant physiological indices are highly correlated mutually. The phenotype of shorter root length and premature senescence was exhibited in the *35S::GhNAC82* overexpressing Arabidopsis compared with wild-type. Lines of *GhNAC82*-VIGS (virus-induced gene silencing) were showed stronger vegetative growth compared to the negative control in premature senescence cotton. Based on the above results, we reasoned that the *GhNAC82* is a negative regulator in plant growth and developmental processes, transgenic plants overexpressing showed early senescence, and gene silencing led to delayed senescence.

## Data Availability

The data presented in this study are available within the article.

## Supplementary Materials

The following supporting information can be downloaded at: https://www.mdpi.com/article/10.3390/plants11111491/s1. Table S1: List of the primers used; Table S2: Gene information of *GhNAC82* and family members in upland cotton; Table S3: The cis -acting regulatory elements of the *GhNAC82* promoter region.
